# Preconditioning diabetic mesenchymal stem cells with myogenic medium increases their ability to repair diabetic heart

**DOI:** 10.1186/scrt207

**Published:** 2013-05-24

**Authors:** Mohsin Khan, Fatima Ali, Sadia Mohsin, Shoaib Akhtar, Azra Mehmood, Mahmood S Choudhery, Shaheen N Khan, Sheikh Riazuddin

**Affiliations:** 1 National Center of Excellence in Molecular Biology, University of the Punjab, 87-West Canal Bank Road, Lahore, Pakistan

**Keywords:** Mesenchymal stem cells, Diabetic heart, Preconditioning, Oxidative stress

## Abstract

**Introduction:**

Mesenchymal stem cells (MSCs) have the potential for treatment of diabetic cardiomyopathy; however, the repair capability of MSCs declines with age and disease. MSCs from diabetic animals exhibit impaired survival, proliferation, and differentiation and therefore require a strategy to improve their function. The aim of the study was to develop a preconditioning strategy to augment the ability of MSCs from diabetes patients to repair the diabetic heart.

**Methods:**

Diabetes was induced in C57BL/6 mice (6 to 8 weeks) with streptozotocin injections (55 mg/kg) for 5 consecutive days. MSCs isolated from diabetic animals were preconditioned with medium from cardiomyocytes exposed to oxidative stress and high glucose (HG/H-CCM).

**Results:**

Gene expression of *VEGF*, *ANG-1*, *GATA-4*, *NKx2.5 MEF2c, PCNA,* and *eNOS* was upregulated after preconditioning with HG/H-CCM, as evidenced by reverse transcriptase/polymerase chain reaction (RT-PCR). Concurrently, increased AKT phosphorylation, proliferation, angiogenic ability, and reduced levels of apoptosis were observed in HG/H-CCM-preconditioned diabetic MSCs compared with nontreated controls. HG/H-CCM-preconditioned diabetic-mouse-derived MSCs (dmMSCs) were transplanted in diabetic animals and demonstrated increased homing concomitant with augmented heart function. Gene expression of angiogenic and cardiac markers was significantly upregulated in conjunction with paracrine factors (*IGF-1, HGF, SDF-1, FGF-2*) and, in addition, reduced fibrosis, apoptosis, and increased angiogenesis was observed in diabetic hearts 4 weeks after transplantation of preconditioned dmMSCs compared with hearts with nontreated diabetic MSCs.

**Conclusions:**

Preconditioning with HG/H-CCM enhances survival, proliferation, and the angiogenic ability of dmMSCs, augmenting their ability to improve function in a diabetic heart.

## Introduction

Diabetes mellitus increases the incidence of heart failure and mortality in diabetes patients [1]. Diabetic cardiomyopathy is associated with diastolic and systolic impairments, leading to endothelial dysfunction, alterations in glucose metabolism, increased fatty acid oxidation, and generation of free radicals [2-4]. Endothelial dysfunction significantly alters angiogenesis in a diabetic heart because of impaired nitric oxide production [5] that further contributes to the etiology of the disease. Restoration of neoangiogenesis within a diabetic heart remains a desirable treatment modality and may augment the depleted diabetic heart function.

Mesenchymal stem cells (MSCs) exhibit multilineage differentiation potential [6-8] and have been widely used in cell-based therapies for the alleviation of a number of impairments, including cardiovascular disorders [9,10]. Even though MSCs represent a feasible choice for myocardial repair, recent evidence implies that the capability of MSCs to repair damaged tissues declines with age and disease [11-14]. A number of preconditioning strategies have been used to enhance depleted stem cell function by treatment with growth factors, hypoxic shock, and antiaging compounds [15,16]. Interestingly, hypoxic preconditioning has been shown to increase mobilization, adhesion, survival, and cardiomyogenic differentiation of MSCs [17-19]. In addition, the age-depleted angiogenic ability of MSCs can be enhanced by hypoxic preconditioning, leading to improved therapeutic efficiency of aged MSCs [20].

Onset of diabetes leads to premature aging of stem cells [21] and is associated with impaired proliferation, paracrine ability, and myogenic differentiation of bone marrow-derived MSCs [22]. Diabetic MSCs demonstrate low differentiation ability in culture conditions, yet preconditioning strategies have not been exploited to improve their function and thus represent an area of further research. In the present study, we demonstrated that diabetic-mouse MSCs (dmMSCs) cultured under high glucose and oxidative stress have increased survival, proliferation, and angiogenic ability. Furthermore, adoptive transfer of preconditioned dmMSCs leads to significant improvement in diabetic heart function.

## Materials and methods

### Animals

The animals used in this study were treated as per *Instructions of the Care and Use of Laboratory Animals* published by the U.S. National Institutes of Health (NIH Publication No. 85–23, revised 1985), and the study was approved by the Institutional Review Committee at the National Center of Excellence in Molecular Biology, Lahore, Pakistan.

### Cell culture

MSCs were isolated and cultured as described [23]. In brief, 60 days after diabetes induction, animals were anesthetized with a single intraperitoneal pentobarbital (40 mg/kg) injection. MSCs from tibias and femurs of C57BL/6 mice were isolated and cultured in Iscove Modified Dulbecco Medium (IMDM) supplemented with 20% fetal bovine serum (FBS), penicillin (100 U/ml), and streptomycin (100 μg/ml). Sprague–Dawley rat pups (*n* = 10) were anesthetized on ice followed by whole-heart removal for cardiomyocyte isolation by using a cardiomyocytes isolation system (Worthington Biomedical Corporation, NJ USA), as described previously [14].

### Oxidative stress

Cardiomyocytes were divided into four experimental treatment groups: (I) low glucose (LG; glucose, 5 m*M*); (II) low glucose H_2_O_2_ (LG/H); (III) high glucose (HG; glucose, 25 m*M*); and (IV) high glucose H_2_O_2_ (HG/H). Oxidative stress was induced with H_2_O_2_ (100 μ*M*) for 60 and 90 minutes in serum-free medium. Cardiomyocytes kept in glucose and serum-free medium were used as the control.

### Enzyme-linked immunosorbent assay

VEGF concentration was determined by using VEGF ELISA kit (Invitrogen, CA USA), according to the manufacturer’s protocol. Medium from cardiomyocyte cultures after no treatment and oxidative stress was collected and centrifuged at 2,000 *g* for 10 minutes at room temperature.

### Preconditioning of diabetic-mouse-derived MSCs

Preconditioning of diabetic-mouse-derived MSCs (dmMSCs) was done by using two strategies. In the first strategy, dmMSCs were preconditioned with cardiomyogenic medium (CCM) comprising the four treatments described that is, (I) LG, (II) LG/ H, (III) HG, and (IV) HG/H.

In the second strategy, we used VEGF pretreatment in concentrations corresponding to ELISA readouts in four CCM treatment groups with the aim to differentiate the effect of cardiomyogenic medium from the solitary effect of VEGF on dmMSCs function. VEGF concentrations in the four groups were as follows: (I) 0.1 ng/ml, (II) 0.05 ng/ml (II), (III) 0.065 ng/ml, and (IV) 0.145 ng/ml. Preconditioning was done for 48 hours, and nontreated dmMSCs were used as controls.

### Gene-expression profiling

RNA was extracted from normal MSCs, nontreated dmMSCs, preconditioned dmMSCs, and heart tissue samples by using Trizole reagent (Invitrogen, USA) and quantified with an ND-1000 spectrophotometer (NanoDrop Technologies, DE USA). cDNA synthesis was performed from 1 μg RNA by using a reverse transcriptase kit (Invitrogen). Real-time RT-PCR was carried out by using SYBR Green PCR Super Mix (BioRad, CA USA), 8 m*M* of each primer, and 100 to 500 ng/ml of template cDNA on BioRad System iQ5. The relative ratio and standard deviation between the normal and treated samples were calculated by using the comparative Ct method (DD Ct value), as recommended by the BioRad iQ5system. RT-PCR analysis was carried out for *IGF-1, p16INK4a, p66shc, p53, VEGF*, *ANG-1*, *NF-κB*, *MEF2c*, *GATA-4*, *NKx2.5*, *CD31*, *CD34*, *PCNA, eNOS,* and *iNOS* on a GeneAmp PCR system 9700 (Applied Biosystems, CA USA). *GAPDH* was used as an internal control, and bands were quantified by using ImageJ. The sequences (5′ to 3′) for the primer pairs and their product lengths (bp) are mentioned in Table 1.

**Table 1:** Primer sequences

**Genes**	**Product size (bp)**	**Sequences (5′ to 3′)**
GAPDH (f)	370	CTCTTGCTCTCAGTATCCTTG
GAPDH (r)		GCTCACTGGCATGGCCTTCC
IGF-1 (f)	166	AGGCTATGGCTCCAGCATTC
IGF-1 (r)		AGTCTTGGGCATGTCAGTGTC
HGF (f)	167	TCACACAGAATCAGGCAAGACT
HGF (r)		AAGGGGTGTCAGGGTCAA
SDF-1 (f)	122	CGGCTGAAGAACAACAACAG
SDF-1 (r)		GGGGGTCTACTGGAAAGTCC
FGF-2 (f)	154	TGTCTATCAAGGGAGTGTGTGC
FGF-2 (r)		CAACTGGAGTATTTCCGTGACC
ANG1 (f)	143	GACACCTTGAAGGAGGAGAAAG
ANG1 (r)		GTGTCCATGAGCTCCAGTTGT
VEGF (f)	200	ACCCCGACGAGATAGAGTACAT
VEGF (r)		CTTCTAATGCCCCTCCTTGT
GATA-4 (f)	90	CCTCTCCCAGGAACATCAAA
GATA-4 (r)		ACCCATAGTCACCAAGGCTG
NKx 2.5 (f)	147	GGTCTCAATGCCTATGGCTAC
NKx 2.5 (r)		GTTCACGAAGTTGCTGTTGG
MEF2c (f)	178	CACGCCTGTCACCTAACATCC
MEF2c (r)		TGTTAGCTCTCAAACGCCACAC
PCNA (f)	110	TGACCCTCACCGATACAACA
PCNA (r)		CTGTACAGCACAGCCACGTT
p53 (f)	157	AGCATCTTATCCGGGTGGAAG
p53 (r)		CCCATGCAGGAGCTATTACACA
CD31 (f)	120	CCCAGTGACATTCACAGACA
CD31 (r)		ACCTTGACCCTCAGGATCTC
CD34 (f)	200	GGGTAGCTCTCTGCCTGATG
CD34 (r)		TCTCTGAGATGGCTGGTGTG
NF-κB (f)	200	GCACCTGTTCCAAAGAGCAC
NF-κB (r)		GTGGAGTGAGACATGGACACAC
iNOS (f)	116	GCTACACTTCCAACGCAACA
iNOS (r)		CATGGTGAACACGTTCTTGG
eNOS (f)	123	TGACCCTCACCGATACAACA
eNOS (r)		CTGTACAGCACAGCCACGTT

### Analysis of preconditioned diabetic-mouse-derived MSCs

Two groups of preconditioned dmMSCs were selected for further analysis on the basis of RT-PCR results (that is, HG/H-CCM and the VEGF (0.065 ng/ml) groups.

#### Cell viability

Viability of dmMSCs after preconditioning was done with the trypan blue exclusion method. The preconditioned (CCM and VEGF preconditioned) diabetic cells and diabetic nontreated cells were trypsinized, centrifuged, and counted by using a hemocytometer.

#### Lactate dehydrogenase assay

LDH assay was used to determine the extent of cell damage in both preconditioned groups by using the LDH assay kit (Sigma T0X-7, MO USA) according to the manufacturer’s instructions. Absorbance values were measured by using Spectra max PLUS 384 (Molecular Devices, CA USA) at 490 nm.

#### Apoptosis

Apoptosis was measured in nontreated and preconditioned dmMSCs by using the Phycoerythrin Annexin-V kit (Abcam, CA USA). In brief, 6 × 10^4^ diabetic MSCs were plated in a 24-well plate (Corning, MA USA) and treated with HG/H-CCM and VEGF (0.065 ng/ml) groups followed by H_2_O_2_ (100 μ*M*) treatment for 90 minutes to induce apoptosis. The Annexin-V^+^ cells were then counted at random in five fields per triplicate well for each treatment.

#### Immunoblot

Total protein was extracted from both preconditioned groups and nontreated dmMSCs by using RIPA buffer and estimated with Bradford protein assay (Bio-Rad, CA USA) followed by separation on 10% SDS-PAGE. The membrane was then incubated overnight at 4°C with AKT (Santa Cruz, CA USA), phospo-AKT serine 473 (Abcam), and β-actin (Abcam, CA USA). Anti-mouse and anti-rabbit secondary antibodies (Abcam) HRP-conjugated were applied for 1 hour, and bands were visualized with an enhanced chemiluminescence (ECL) detection system (Amersham Pharmacia Biotech, PE USA).

#### Cell proliferation

A cell-proliferation assay was carried out with a XTT (sodium 3–[1- {phenylaminocarbonyl}-3,4-tetrazolium]-bis{4-methoxy-6-nitro} benzene sulfonic acid hydrate) cell proliferation kit, according to the manufacturer’s protocol (Roche, IN USA). In brief, 4 × 10^3^ dmMSCs were plated in triplicate in a 96-well plate (Corning, USA) overnight in IMDM supplemented with 20% FBS in an incubator at 37°C and 5% CO_2_. Absorbance was taken at 450 nm by using Spectra max PLUS 384 (Molecular Devices), with 650 nm as the reference wavelength.

#### In vitro tube-forming assay

*In vitro* angiogenic potential of both preconditioned groups and nontreated dmMSCs was assessed with a gelatinous protein mixture (Matrigel) assay (BD Bioscience, CA USA), as described previously [23]. In brief, 8 × 10^3^ cells from each treatment group were plated on Matrigel-coated wells and incubated at 37°C in 5% CO_2_. The formation of tubular network was examined after 48 hours. The images were taken in a phase-contrast microscope (IX-51, Olympus, Tokyo Japan) equipped with a digital camera, DP-71 (Olympus).

#### Nitric oxide activity

NOS activity was determined with an NOS colorimetric assay kit (Oxford Biomedical Research, NB 78, Upper Heyford, UK) according to the manufacturer’s instructions. In brief, protein was isolated by using lysis buffer from both preconditioned groups and nontreated dmMSCs to measure NOS activity. Absorbance values were measured by using Spectra max PLUS 384 (Molecular Devices) at 540 nm.

### Diabetic cardiomyopathy model for cell transplantation

Diabetes was induced in C57BL/6 male mice by streptozotocin administration, as described previously [23]. In brief, diabetes was induced in C57BL/6 wild-type mice (6 to 8 weeks old) by intraperitoneal injection of streptozotocin (55 mg/kg) for 5 consecutive days. Because streptozotocin metabolizes within 24 hours, mice were housed with absorbent bedding and food and water *ad libitum*. At day 16 after the initial injection, mice were weighed, and their blood glucose level was measured. Only streptozotocin-induced diabetic mice with blood glucose levels >300 mg/dl were included in the study. Mice after 2 months of diabetes induction were divided into four groups (*n* = 6). Mice in group I served as normal control; group II, as diabetic control injected with saline; group III, as diabetic animals transplanted with nontreated dmMSCs; and group IV, as diabetic animals transplanted with preconditioned dmMSCs. In brief, mice were anesthetized with a single intraperitoneal injection of pentobarbital, and the adequacy of anesthesia was monitored by animal-reflex responses to stimuli. The heart was exposed by left-side limited thoracotomy, and cells were transplanted into the left ventricle. Mice were given subcutaneous buprenorphine (0.1 mg/kg) injections before the procedure and 24 hours after surgery. Cell concentration used was 1 × 10^6^/ml, and animals were given intramyocardial injections of MSCs (10 μl ~ 100,000 cells/animal) at two distinct sites in the left ventricle.

### Assessment of heart function

Mice were anesthetized with sodium pentobarbital (40 mg/kg), and hemodynamic function was assessed by using the Millar apparatus (Millar Instruments, Inc., Houston, TX, USA), as described previously [24]. Left ventricle (LV) systolic function was evaluated with P_max_, dp/dt_max_, and arterial elastance. LV diastolic function was evaluated with end-diastolic pressure and dp/dt_min_. Analysis was carried out by using the Millar PVAN software (version 3.3).

### Fibrosis

Histologic analysis was performed to determine collagen deposition in the diabetic heart. Heart sections were stained with Masson trichrome stain, and 10 random fields were selected and photographed with light microscopy at 200× magnification. The extent of fibrosis in the respective images was quantified by using Image J analysis software.

### Immunohistochemistry

Mice were anesthetized and killed by cervical dislocation 4 weeks after MSC injections. The hearts were removed, and sections were analyzed for the detection of cleaved caspase-3 and eNOS through application of enzyme-labeled polymer (Vector ImmPRESS kit; Vector Laboratories, CA USA). The frozen tissue was cut into 5-μm sections, fixed with 4% formaldehyde, and permeabilized by using 0.2% Triton X-100. Sections were treated with 3% H_2_O_2_ for 10 minutes to block endogenous peroxidase activity, followed by blocking in 5% donkey serum for 1 hour at room temperature. Sections were incubated overnight with primary antibodies of polyclonal rabbit anti-caspase-3 antibody (Santa Cruz Biotechnology) and a rabbit polyclonal anti-eNOS antibody (Abcam) and treated with enzyme horseradish peroxidase (HRP)-conjugated specific secondary antibodies. Sections were incubated with chromogen substrate 3,3′-diaminobenzidine tetrahydro-chloride (DAB) solution and counterstained with the Weigert Iron Hematoxylin solution to stain the nuclei. Capillary density was measured by staining sections from control and transplanted groups with α-smooth muscle actin (α-SMA) (Sigma-Aldrich, MO USA), and small arterioles per five random fields of each section were counted.

### Statistical analysis

Statistical analysis was performed with Graph Pad Prism-5 software and SPSS software. All data values are expressed as mean ± SEM. The comparison between more than two parameters was assessed with ANOVA with the Bonferroni *post hoc* test. The comparison between two groups was performed by using a Student *t* test. *P* < 0.05 was considered significant.

## Results

### Preconditioning of diabetic-mouse-derived MSCs

**Figure 1:**
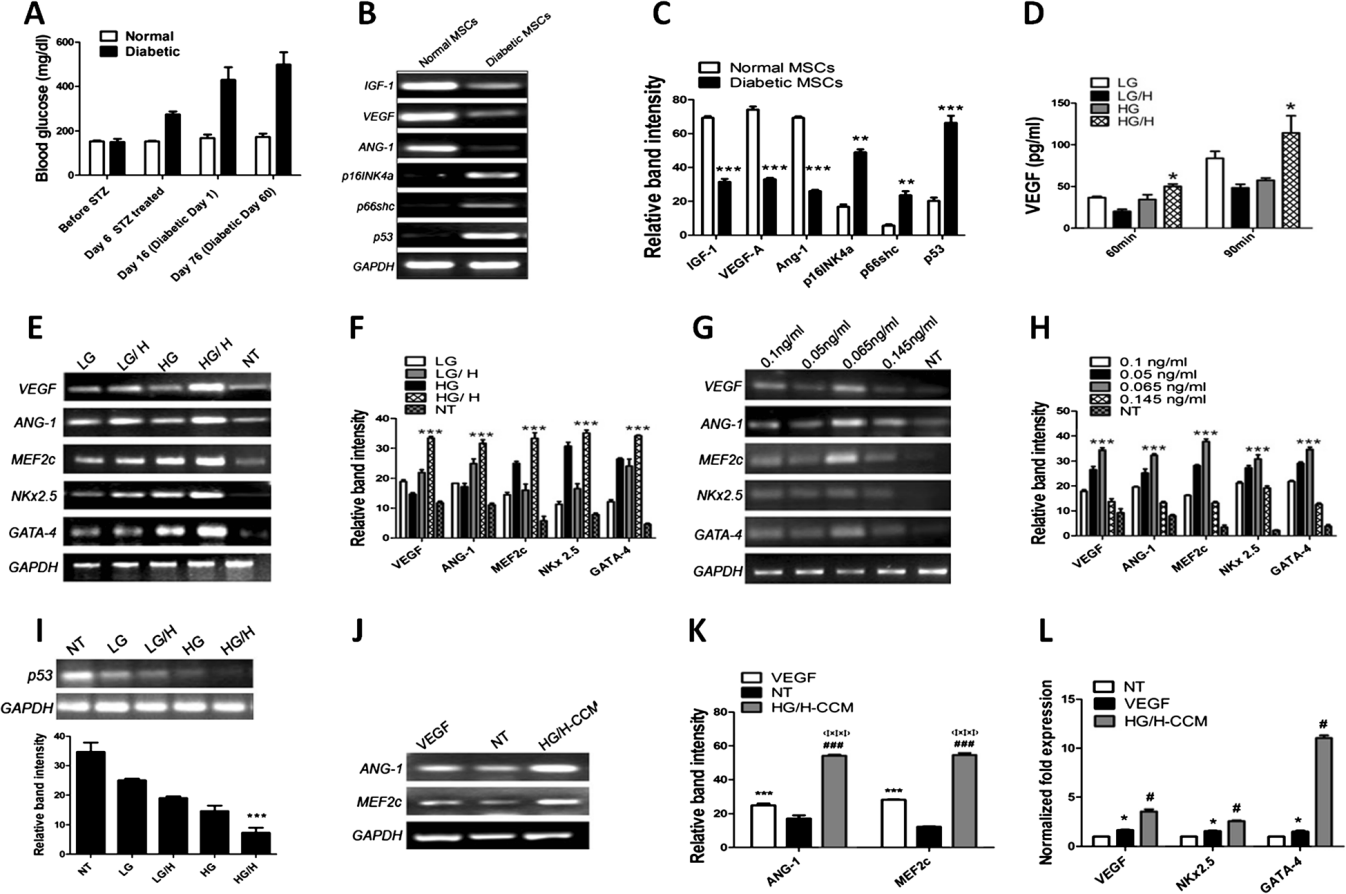
**Preconditioning strategy for the treatment of dmMSCs. (A)** Blood glucose levels during streptozotocin administration until 60 days in C57BL/6 wild-type mice. **(B)** Gene-expression profiling of normal and diabetic MSCs with RT-PCR. **(C)** Gel band quantification by Image J. *P < 0.05; **P < 0.01; and ***P < 0.001 significance for normal MSCs versus diabetic MSCs. **(D)** VEGF release in cardiomyocytes subjected to 60 and 90 minutes of H_2_O_2_-induced oxidative stress combined with varying glucose concentrations, as measured with ELISA. **(E, F)** Gene-expression analysis of dmMSCs preconditioned with media from cardiomyocytes subjected to no treatment or H_2_O_2_ treatment in the presence of low or high glucose. **(G, H)** Gene expression of dmMSCs preconditioned with different concentrations of VEGF. **(I)** Gene-expression analysis of *p53* between different CCM-treated groups compared with nontreatment (NT) along with corresponding quantification. NT versus HG/H, *P < 0.05; **P < 0.01; and ***P < 0.001). **(J, K)** Comparison of gene expression between dmMSCs preconditioned with HG/H-CCM or VEGF (0.065 ng/ml). **(L)** Analysis of gene expression for *VEGF, NKx2.5,* and *GATA-4* by using real-time quantitative PCR. NT versus VEGF, *P < 0.05; **P < 0.01; and ***P < 0.001; NT versus HG/H-CCM, ^#^*P* < 0.05; ^##^*P* < 0.01; and ^###^*P* < 0.001; VEGF versus HG/H-CCM, ^φ^*P* < 0.05; ^φφ^*P* < 0.01; and ^φφφ^*P* < 0.001.

Diabetic-mouse-derived MSCs (dmMSCs) were isolated from diabetic mice 60 days after induction of diabetes and confirmed by blood glucose levels >300 mg/dl (Figure 1A). Characterization of dmMSCs demonstrated increased levels of *p16INK4a, p66shc,* and *p53* concomitant with decreased expression of *IGF-1, VEGF,* and *Ang-1*, compared with normal MSCs, as evidenced by RT-PCR analysis (Figure 1B, C). The dmMSCs were preconditioned with Conditioned Cardiomyogenic Medium (CCM) to improve dmMSCs function. CCM was prepared by induction of oxidative stress in neonatal rat cardiomyocytes (NRCMs) in combination with low glucose (5.5 m*M*) and high glucose (25 m*M*). Results showed that VEGF release increased significantly after 90 minutes of H_2_O_2_ induced oxidative stress compared with 60 minutes in NRCMs (Figure 1D). In contrast, VEGF levels were not detected in nontreated cardiomyocytes kept in glucose and serum-free medium (data not shown). Because 90 minutes of oxidative stress resulted in maximal VEGF release, it was selected as the optimal treatment to be used in combination with high and low glucose for subsequent experiments.

Alternatively, to determine whether VEGF alone can be used as a comparable preconditioning strategy to treatment with CCM, dmMSCs were preconditioned with various concentrations of VEGF corresponding to VEGF ELISA readout after exposure of NRCMs to various treatments, as described earlier.

Gene-expression analysis of angiogenic and cardiac markers was done for all groups of CCM- and VEGF-treated dmMSCs by RT-PCR, whereas nontreated dmMSCs were used as controls. Elevated levels of *VEGF*, *ANG-1* (angiogenic markers), *MEF2c, NKx2.5,* and *GATA-4* (cardiac transcription factors) were observed in the HG/H-CCM group (Figure 1E, F) compared with all other CCM treatment groups. Among the VEGF treatment groups, 0.065 ng/ml VEGF concentration resulted in significantly higher levels of the same angiogenic markers and cardiac transcription factors mentioned earlier compared with other VEGF concentrations and the diabetic nontreated group (Figure 1G, H). Furthermore, a significant decline in p53 expression was observed in dmMSCs treated with HG/H-CCM compared with all other treatment groups (Figure 1I). Determine the best preconditioning strategy, dmMSCs were treated with HG/H-CCM or 0.065 ng/ml VEGF and analyzed with RT-PCR for angiogenic and cardiac-specific markers. Gene-expression analysis results indicated a significant increase in *VEGF*, *ANG-1, MEF2c, NKx2.5,* and *GATA-4* after treatment with HG/H-CCM compared with 0.065 ng/ml VEGF and the diabetic nontreated group (Figure 1J to L).

### CCM preconditioning promotes survival and proliferation of dmMSCs

**Figure 2:**
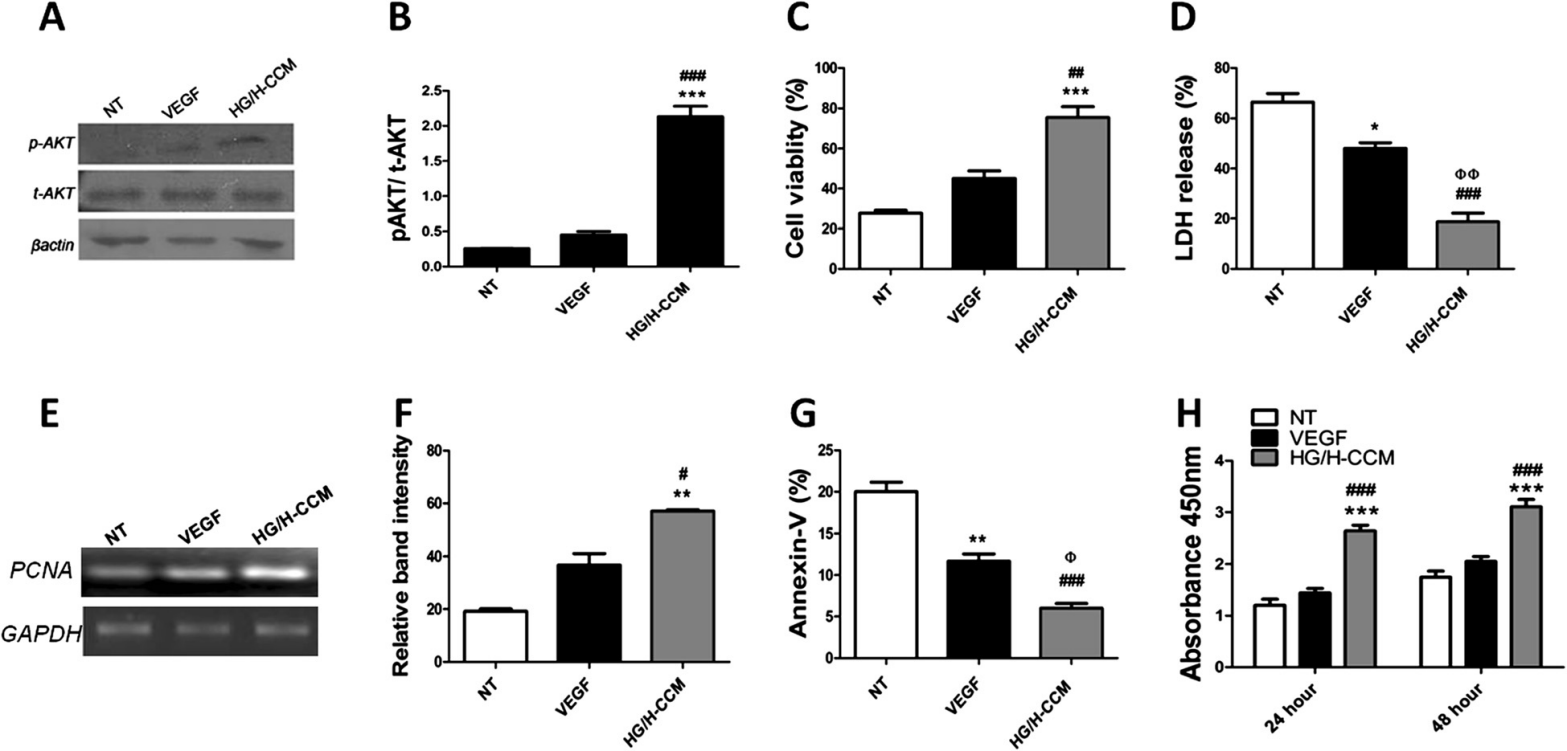
**Increased survival and proliferation of dmMSCs after preconditioning. (A, B)** Increased *AKT* phosphorylation in dmMSCs treated with HG/H-CCM compared with VEGF (0.065 ng/ml). **(C)** Trypan blue viability assay. NT versus HG/H-CCM *P < 0.05; **P < 0.01; and ***P < 0.001; VEGF versus HG/H-CCM ^#^*P* < 0.05; ^##^*P* < 0.01; and ^###^*P* < 0.001. **(D)** LDH-release assay. **(E)***PCNA* gene expression after preconditioning with HG/H-CCM and VEGF (0.065 ng/ml). **(F)** Quantification of *PCNA* gel bands was done by using Image J software. **(G)** Quantification of Annexin-V (%) in nontreated, HG/H-CCM and VEGF (0.065 ng/ml) preconditioned dmMSCs along with quantification. NT versus VEGF, *P < 0.05; **P < 0.01; and ***P < 0.001; NT versus HG/H-CCM, ^#^*P* < 0.05; ^##^*P* < 0.01; and ^###^*P* < 0.001; VEGF versus HG/H-CCM, ^φ^*P* < 0.05; ^φφ^*P* < 0.01; and ^φφφ^*P* < 0.001. **(H)** XTT cell-proliferation assay confirms increased proliferation after HG/H-CCM treatment compared with VEGF (0.065 ng/ml) or nontreated control cells. NT versus HG/H-CCM, *P < 0.05; **P < 0.01; and ***P < 0.001; HG/H-CCM versus VEGF, ^#^*P* < 0.05; ^##^*P* < 0.01; and ^###^*P* < 0.001.

The effect of preconditioning on survival was analyzed with immunoblot for *AKT* and phospho*AKT*. *In vitro* experiments showed that the HG/H-CCM preconditioned group significantly increased *AKT* phosphorylation compared with VEGF (0.065 ng/ml) or nontreated dmMSCs (Figure 2A, B). Increased cell viability was observed in HG/H-CCM (76% ± 5.54%) compared with VEGF (45% ± 3.7%) and nontreated dmMSCs (28% ± 1.4%), evidenced by the trypan-blue exclusion assay (Figure 2C). Cellular damage evidenced by the LDH-release assay was significantly reduced after HG/H-CCM (18% ± 3.5%) treatment compared with VEGF (48% ± 2.3%) and nontreated dmMSCs (66% ± 3.4%) (Figure 2D). Concurrently, a significantly low number of Annexin-V^+^ cells was observed in HG/H-CCM (6% ± 1.88%) compared with VEGF (9% ± 1.4%) and nontreated dmMSCs (20% ± 6.1%) (Figure 2G).

Gene expression of *PCNA* was observed to be significantly increased after HG/H-CCM treatment versus VEGF versus nontreated dmMSCs (Figure 2E, F). In addition, cell proliferation was assessed after treatment with HG/H-CCM and VEGF (0.065 ng/ml). Preconditioning with HG/H-CCM resulted in significantly higher proliferation at 24 and 48 hours (2.61 ± 0.10; 3.1 ± 0.14) followed by VEGF treatment (1.44 ± 0.84; 2.0 ± 0.96) and nontreated dmMSCs (1.2 ± 0.11; 1.74 ± 0.12) (Figure 2H).

### Increased angiogenic ability of dmMSCs after CCM preconditioning

**Figure 3:**
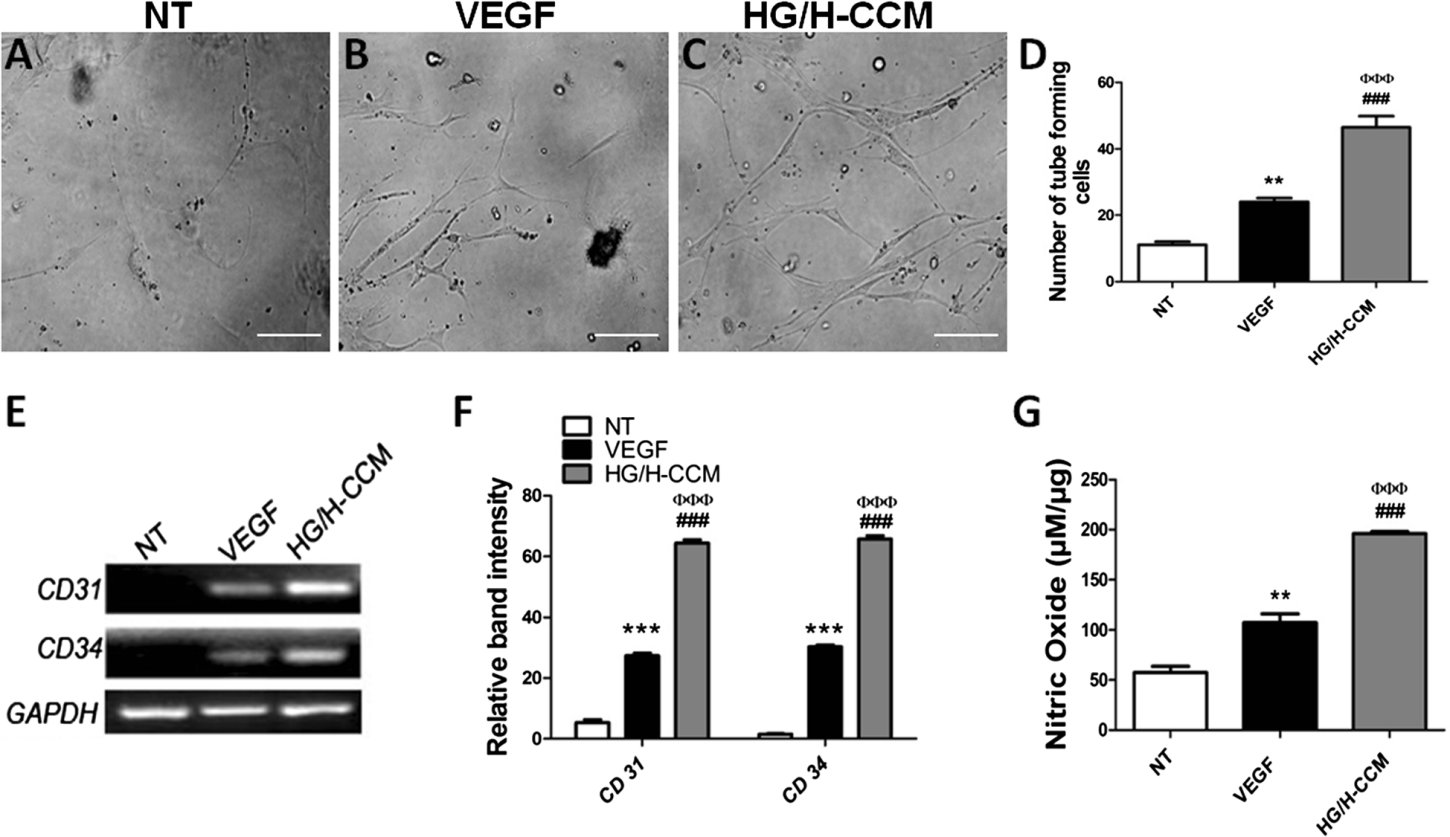
**Effect of preconditioning on the angiogenic ability of dmMSCs. (A-D)** Matrigel assay of preconditioned and nontreated dmMSCs. Images show increased tube formation after HG/H-CCM group versus VEGF (0.065 ng/ml) versus nontreated dmMSCs. Scale bar about 20 μm. **(E, F)** Gene expression of *CD31* and *CD34* endothelial lineage markers. Quantification of gel bands by using Image J software. **(G)** NOS activity of dmMSCs preconditioned with HG/H-CCM or VEGF. NT versus VEGF, *P < 0.05; **P < 0.01; and ***P < 0.001; NT versus HG/H-CCM, ^#^*P* < 0.05; ^##^*P* < 0.01; and ^###^*P* < 0.001; VEGF versus HG/H-CCM, ^φ^*P* < 0.05; ^φφ^*P* < 0.01; and ^φφφ^*P* < 0.001.

The effect of HG/H-CCM or VEGF preconditioning on the ability of dmMSCs for tube formation was assessed by their culture on Matrigel. The *in vitro* tube-formation ability of dmMSCs was significantly improved in the HG/H-CCM-conditioned group compared with VEGF (0.065 ng/ml) and nontreated dmMSCs (Figure 3A through D). Significant upregulation of angiogenic markers *CD31* and *CD34* was observed in the HG/H-CCM group compared with VEGF and nontreated dmMSCs, as evidenced by gene-expression analysis (Figure 3E, F). Concurrently, dmMSCs showed significantly augmented NOS activity after 48 hours of preconditioning in HG/H-CCM (196 ± 2.0 μ*M*) treatment compared with VEGF (107 ± 8.8 μ*M*) and nontreated dmMSCs (57 ± 6.3 μ*M*) (Figure 3G).

### Increased homing and paracrine signaling

**Figure 4:**
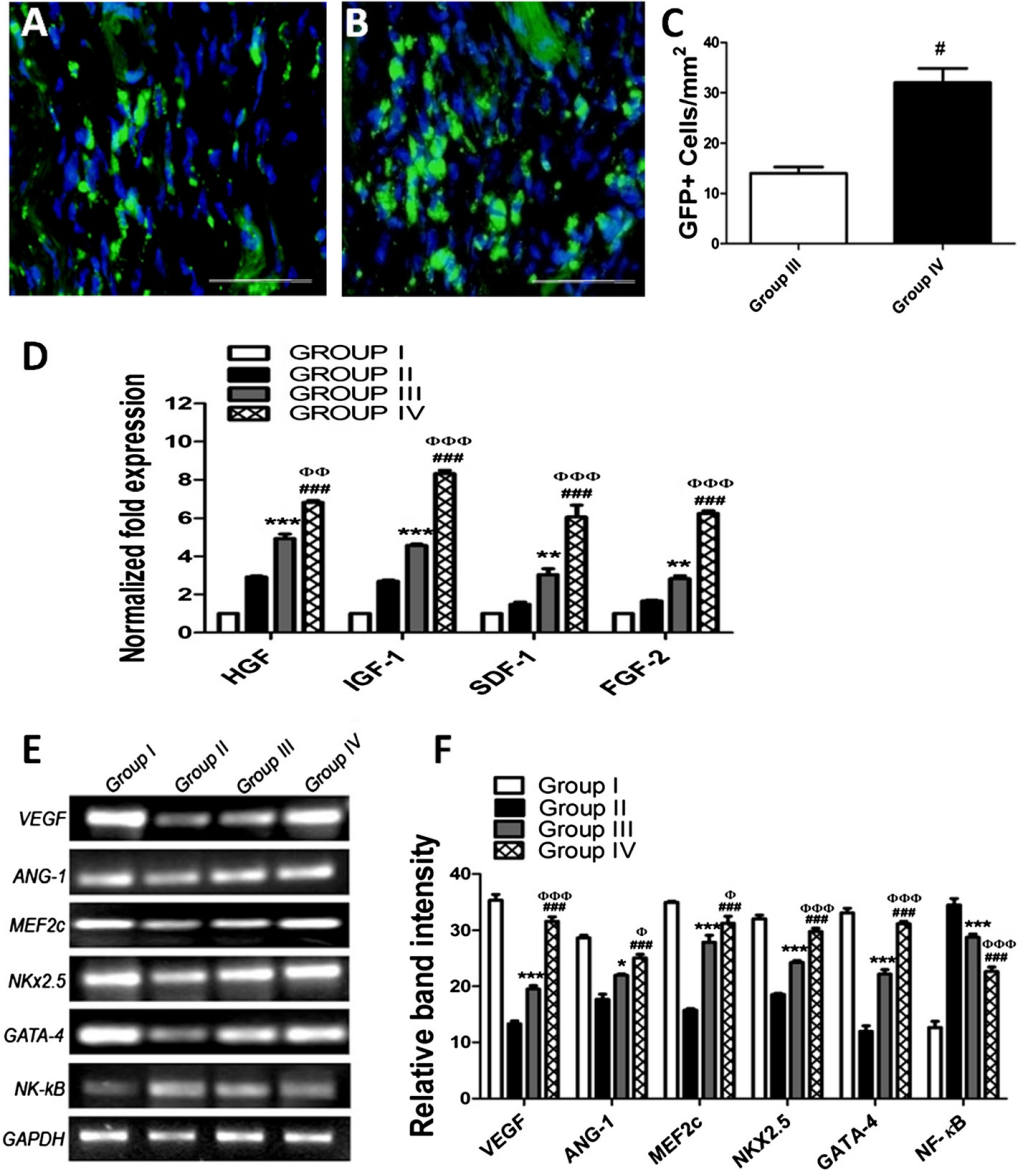
**Increased homing and paracrine signaling of preconditioned dmMSCs. (A, B)** Increased homing of preconditioned dmMSCs compared with nontreated MSCs along with corresponding quantification in **(C);** Scale bar about 20 μm. **(D)** Gene-expression analysis of paracrine factors in all animal groups by using quantitative real-time PCR. **(E)** Gene-expression analysis in hearts from all animal groups. **(F)** Gel quantification using image J. Group II versus group III, *P < 0.05; **P < 0.01, and ***P < 0.001; group II versus group IV, ^#^*P* < 0.05; ^##^*P* < 0.01; and ^###^*P* < 0.001; group III versus group IV, ^φ^*P* < 0.05; ^φφ^*P* < 0.01; and ^φφφ^*P* < 0.001.

Increased survival of preconditioned dmMSCs was observed in diabetic animals 4 weeks after transplantation compared with nontreated MSCs, confirming the effect of preconditioning on dmMSC homing ability (Figure 4A to C). Analysis of paracrine factors demonstrated significant upregulation of *IGF-1, FGF-2, SDF-1,* and *HGF* in group IV animals transplanted with dmMSCs preconditioned with HG/H-CCM compared with group III animals transplanted with nontreated dmMSCs (Figure 4D). Furthermore, gene-expression analysis demonstrated that group IV animals, transplanted with dmMSCs preconditioned with HG/H-CCM, exhibited significantly upregulated expression of *VEGF*, *ANG-1*, *MEF2c*, *NKx2.5*, *GATA-4,* and *NF-κB,* as evidenced by RT-PCR compared with group III animals transplanted with nontreated MSCs and diabetic control animals (Figure 4E, F).

### CCM preconditioning improves cardiac function

**Figure 5:**
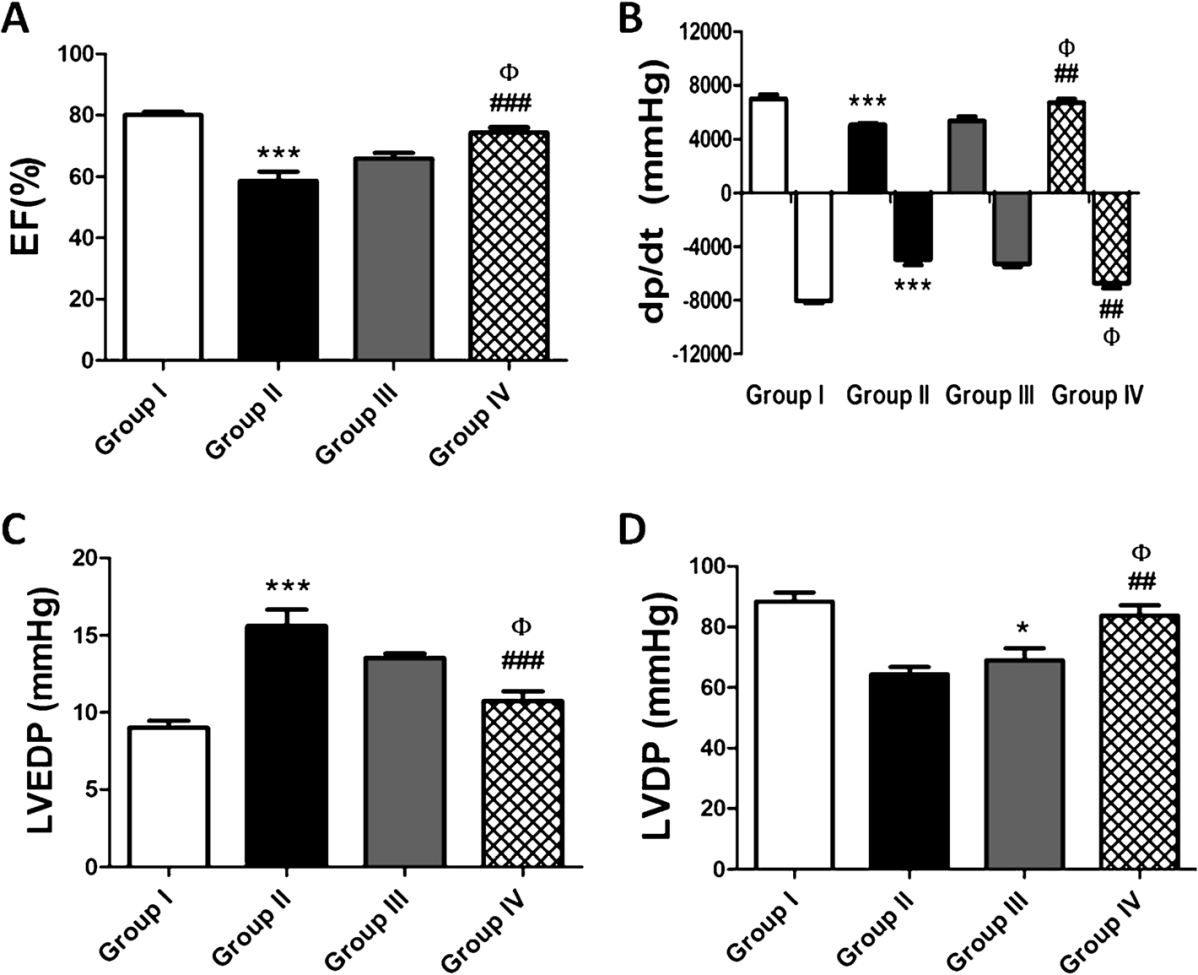
**Cardiac function.** Graphic representation of following parameters: **(A)** Ejection fraction (%) in four groups. **(B)** dp/dt. **(C** and **D)** represent the left ventricle end-diastolic pressure and left ventricle diastolic pressure, respectively. Group II versus group III, *P < 0.05; **P < 0.01; and ***P < 0.001; group II versus group IV, ^#^*P* < 0.05; ^##^*P* < 0.01; and ^###^*P* < 0.001; group III versus Group IV, ^φ^*P* < 0.05; ^φφ^*P* < 0.01; and ^φφφ^*P* < 0.001.

Cardiac function of diabetic mice transplanted with HG/H-CCM preconditioned or nontreated dmMSCs was assessed after 4 weeks with the Millar apparatus (Figure 5A to D). Load-dependent-arameter ejection fraction (EF) revealed marked increases in group IV and group III as compared with group II. LV systolic function, as determined by dp/dt_max_ and dp/dt_min_, was significantly improved in group IV mice transplanted with HG/H-CCM preconditioned MSCs compared with group III mice that received nontreated dmMSCs. Collectively, a marked improvement was observed in the functionality of the diabetic heart after transplantation of the HG/H-CCM-preconditioned MSCs.

### CCM preconditioning enhances angiogenesis

**Figure 6:**
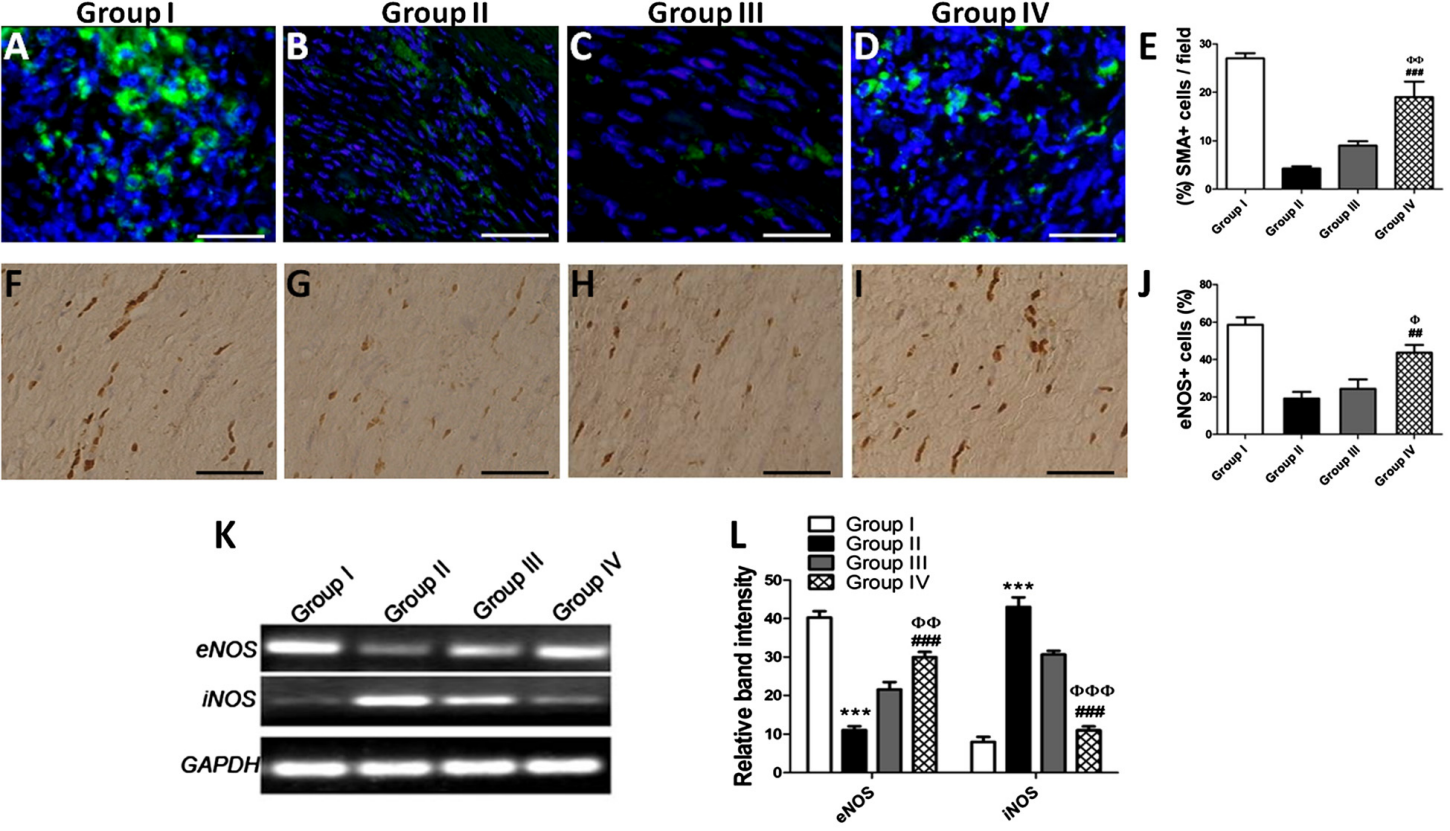
**Preconditioned dmMSCs enhance angiogenesis in diabetic heart. (A** through **D)** Blood-vessel density of cells expressing smooth muscle actin along with corresponding quantification in **(E)**; scale bar about 20 μm. **(F** through **J)** Quantification of eNOS-positive cells in all the groups. Group II versus group IV, ^#^*P* < 0.05; ^##^*P* < 0.01; and ^###^*P* < 0.001; group III versus group IV, ^φ^*P* < 0.05; ^φφ^*P* < 0.01; and ^φφφ^*P* < 0.001. **(K)** Gene-expression analysis of *eNOS* and *iNOS* in all groups with RT-PCR. **(L)** Quantification of gel bands by using Image J software. Group II versus group III, *P < 0.05; **P < 0.01; and ***P < 0.001; Group II versus group IV, ^#^*P* < 0.05; ^##^*P* < 0.01; and ^###^*P* < 0.001; group III versus group IV, ^φ^*P* < 0.05; ^φφ^*P* < 0.01; and ^φφφ^*P* < 0.001.

Diabetic heart transplanted with HG/H-CCM-preconditioned MSCs showed significant improvement in diabetic myocardium. The number of blood vessels (α-SMA positive) was significantly increased in group IV compared with group III mice (21 ± 0.001 versus 13 ± 0.001) (Figure 6A through E). Intense eNOS activity was observed in the cardiac tissue of group IV mice transplanted with HG/H-CCM-preconditioned MSCs, whereas it was much weaker in the group III and II mice. Quantification of eNOS^+^ cells confirmed significantly more cells in diabetic hearts treated with HG/H-CCM-preconditioned MSCs compared with all other groups (Figure 6F through J). Immunostaining results for eNOS were confirmed with RT-PCR and demonstrated increased eNOS and reduced iNOS in group IV animals compared with groups III and II. GAPDH was used as cDNA quality and loading control (Figure 6K through L).

### Effect of CCM preconditioning on fibrosis

**Figure 7:**
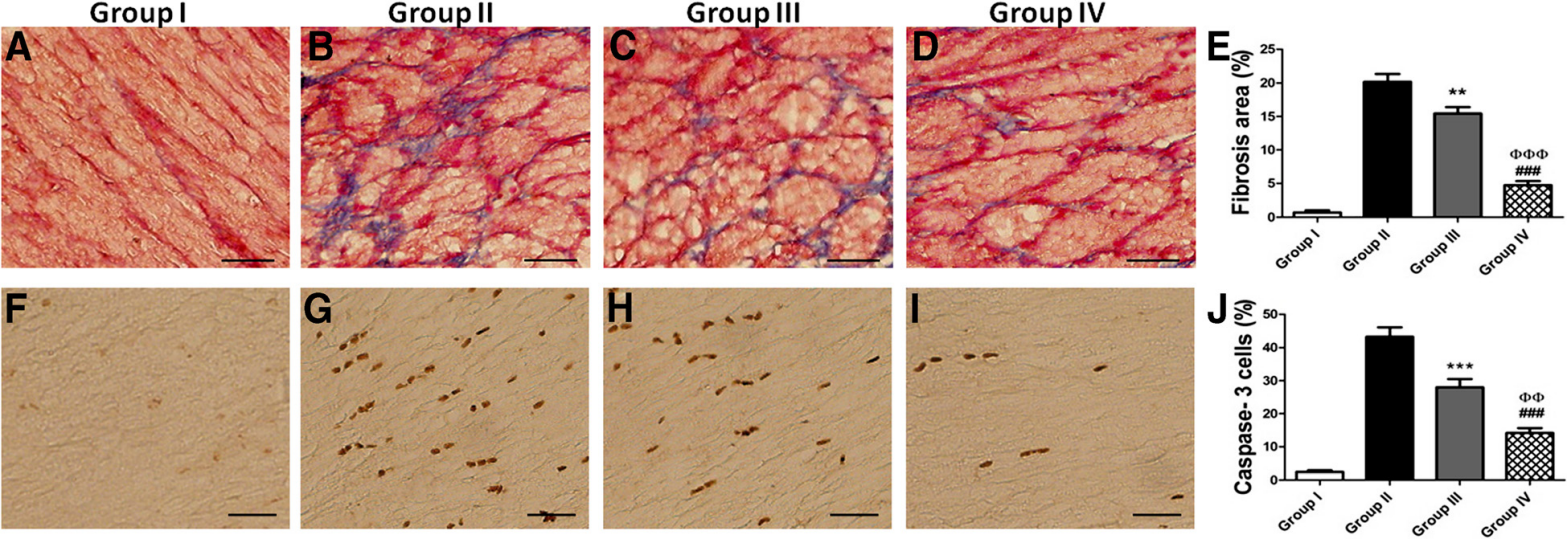
**Decreased fibrosis and apoptosis in diabetic hearts transplanted with preconditioned dmMSCs.** Analysis of fibrosis in **(A)** group I, **(B)** group II, **(C)** group III, and **(D)** group IV with Masson trichrome staining of diabetic heart sections. **(E)** Quantification of fibrosis in all animal groups. Cleaved casapse-3 staining of diabetic heart for the identification of apoptosis. Images represent **(F)** group I; **(G)** group II; **(H)** group III, **(I)** group IV (scale bar about 20 μm). **(J)** Cleaved caspase-3 analysis of apoptosis in all experimental groups. Quantification was done with image J software. Group II versus group III, *P < 0.05; **P < 0.01; and ***P < 0.001; group II versus group IV, ^#^*P* < 0.05; ^##^*P* < 0.01; and ^###^*P* < 0.001; group III versus group IV, ^φ^*P* < 0.05; ^φφ^*P* < 0.01; and ^φφφ^*P* < 0.001.

dmMSCs preconditioned with HG/H-CCM demonstrated reduced fibrosis, as assessed with Masson trichrome staining (Figure 7A through E). Fibrosis 4 weeks after transplantation in animals was significantly lower in group IV (4.7% ± 0.60%) compared with group III (15.4% ± 0.94%) and group II mice (20.1% ± 1.18%).

### Effect of CCM preconditioning on apoptosis

Analysis for apoptosis in all animal groups was done with cleaved caspase-3 staining. Increased caspase-3^+^ cells were observed in group II (43.2% ± 2.85%) compared with group III (28% ± 2.5%) and group IV (14.2% ± 1.46%). As expected, lower apoptotic levels were found in the diabetic myocardium transplanted with HG/H-CCM-preconditioned MSCs (Figure 7E through I).

## Discussion

Diabetes mellitus is a major risk factor for development of myocardial infarction in a large segment of the population, causing an enormous economic burden. A number of studies have shown that diabetic hearts are more sensitive to ischemic insults that can lead to heart failure [25,26]. Development of diabetic cardiomyopathy is associated with decreased diastolic compliance, increased interstitial fibrosis, and myocyte hypertrophy [25]. Accrual of reactive oxygen species (ROS) in a diabetic heart leads to endothelium dysfunction characterized by vascular remodeling, disappearance of capillary endothelium, and altered gene and protein expression in endothelial cells [27]. Cell-based therapies using MSCs have been successfully used for treatment of the damaged heart, yet stem cells isolated from diabetic animals have severely reduced reparability [21], resulting in diminished heart repair after autologous transplantation [28]. Therefore, the present study demonstrated that preconditioning with HG/H-CCM can augment survival, proliferation, and angiogenic ability of MSCs isolated from streptozotocin-induced diabetic mice.

Bone marrow-derived MSCs have been shown to be the best candidates for myocardial regeneration and possess the ability to form cardiomyocytes, endothelial cells, and smooth muscle cells [29,30]. Adoptive transfer of MSCs in a rat model of diabetic cardiomyopathy is associated with enhanced angiogenesis, myogenesis, and cardiac function [31,32]. Nevertheless, the success of MSCs therapy for diabetic heart repair has been accompanied by reports of functional MSC decline as a consequence of diabetes. MSCs from streptozotocin-induced diabetic rats have impaired abilities for proliferation, paracrine, antiapoptosis, and myogenic differentiation [22]. Similarly, the onset of diabetes can attenuate the osteogenic differentiation ability of MSCs [33]. Therefore, augmentation of depleted MSC function becomes imperative if MSCs are to be used for autologous therapy to repair diabetic heart failure.

Preconditioning of stem cells with growth factors [23], hypoxic shock [34], and antiaging [35] compounds represents an effective strategy to enhance survival, proliferation, and differentiation of MSCs. Recent studies showed that ischemic preconditioning is a powerful regulator of cardioprotection [36-38], and we hypothesized that short-term stimulation of cardiomyocytes with a combination of stress stimuli can mimic *in vitro* the effects of ischemic preconditioning observed *in vivo*. Therefore, the normal and diabetic microenvironments of the myocardium were simulated *in vitro* by treating NRCMs with a combination of oxidative stress and high glucose, with the premise that treatment of dmMSCs with HG/H-CCM can enhance survival, proliferation, and importantly, increase the angiogenic ability of dmMSCs. We showed previously that diabetic mouse-derived MSCs have impaired proliferation, antioxidant levels, and survival signaling [23], meriting the need for preconditioning. Increased VEGF levels were observed in conditioned medium from NRCMs treated with HG/H and concurs with previous reports showing upregulation of VEGF as a consequence of hypoxia [34]. In parallel to medium preconditioning, dmMSCs were preconditioned with VEGF alone to highlight that HG/H-CCM may contain other angiogenic and nonangiogenic paracrine factors, and these factors collectively can produce the optimal cellular response compared with treatment with VEGF alone.

dmMSCs were preconditioned with HG/H-CCM in parallel with VEGF. HG/H-CCM preconditioning significantly increased survival and proliferation of dmMSCs compared with treatment with VEGF alone and nontreated control. Our study also shows that the HG/H-CCM preconditioning favors commitment of dmMSCs to the endothelial lineage, as confirmed by RT-PCR expression of endothelial lineage markers *CD31* and *CD34,* concomitant with increased tube formation and nitric oxide production. These results concur with previous reports showing that oxidative stress can enhance recruitment [39], survival [34,40], migration [16], and differentiation [41] of MSCs. Furthermore, it has been shown that oxidative stress can augment the angiogenic potential of age-depleted bone marrow and adipose-derived MSCs [42] and concurs with our observed results showing increased angiogenic ability of diabetic MSCs after preconditioning with HG/H-CCM.

Adoptive transfer of HG/H-CCM-preconditioned dmMSCs in diabetic animals showed augmented cardiac function and was concomitant with reduced collagen deposition and apoptosis, compared with nontreated dmMSCs and diabetic controls. Interestingly, transplantation of HG/H-CCM-preconditioned dmMSCs resulted in significant augmentation of angiogenesis in the diabetic heart, evidenced by increased SMA^+^ cells and eNOS expression, whereas iNOS expression was significantly reduced compared with animals transplanted with nontreated dmMSCs and diabetic controls. Gene-expression analysis of hearts from animals transplanted with HG/H-CCM-preconditioned dmMSCs showed increased angiogenic (*VEGF, ANG-1*) and cardiac (*MEF2c, GATA-4, NKx2.5*) markers but decreased expression of *NF-κB* compared with animals with nontreated dmMSCs and diabetic controls. Conversely, increased paracrine factors (*IGF-1, FGF-2, SDF-1, HGF*) were found in the hearts of animals transplanted with CCM-preconditioned dmMSCs, supporting the idea that preconditioned dmMSCs have better survival, angiogenic ability, and can improve heart function by augmentation of a diabetic environment, possibly through a paracrine-mediated effect.

MSCs represent an attractive option for cell-based therapies for the repair of the diabetic heart. However, MSCs from a diabetic animal have attenuated ability to survive, proliferate, and repair the heart. Therefore, we showed that preconditioning with HG/H-CCM can augment survival, proliferation, and the angiogenic ability of dmMSCs. Furthermore, adoptive transfer of dmMSCs preconditioned with HG/H-CCM improves cardiac function and angiogenesis while reducing fibrosis and apoptosis in the diabetic heart, possibly through a paracrine effect. Thus, preconditioning of dmMSCs represents an effective strategy to improve depleted MSC function and holds promise for the treatment of diabetic heart failure.

## Conclusions

We demonstrated that preconditioning with cardiomyogenic medium can improve survival, proliferation, and cardiac-repair ability of dmMSCs. Development of diabetes mellitus has been shown to attenuate MSC survival, proliferation, and the ability to repair the heart after myocardial infarction. Preconditioning strategies consist of growth factors, hypoxia, and other factors and can augment depleted stem-cell function to repair damaged organs. We used a preconditioning strategy based on medium from cardiomyocytes treated with oxidative stress and high glucose, with the premise that acute exposure to stress can stimulate cardiomyocytes to release paracrine factors in the medium. Preconditioning of dmMSCs with medium rich in paracrine factors from the cardiomyocytes increased survival, proliferation, and angiogenic ability to dmMSCs. Therefore, we report here a viable strategy to enhance dmMSC function and increase repair of diabetic myocardium.

## Abbreviations

CCM: Conditioned cardiomyogenic medium; dmMSCs: diabetic mouse-derived mesenchymal stem cells; dp/dtmax: Maximal change in pressure/change in time; dp/dtmin: Minimal change in pressure/change in time; HG/H: High-glucose/H_2_O_2_; HG/H-CCM: High-glucose/H_2_O_2_-conditioned cardiomyogenic medium; IMDM: Iscove modified Dulbecco medium; LDH: Lactate dehydrogenase; LG/H: Low-glucose/H_2_O_2_; LV: Left ventricle; MSCs: Mesenchymal stem cells; NOS: Nitric oxide synthase; NRCM: Neonatal rat cardiomyocytes; Pmax: Maximal pressure; ROS: Reactive oxygen species; SMA: Smooth muscle actin.

## Competing interests

The authors have no financial conflicts of interest.

## Authors’ contributions

MK carried out cardiomyocyte isolation, participated in study design, and drafted the manuscript. FA performed *in vitro* assays, *in vivo* studies, and drafted the manuscript. SM participated in study design, statistical analysis, and helped draft the manuscript. SA participated in generating the diabetic mouse model. AM carried out microscopy. MSC performed invasive hemodynamic procedures. SNK participated in study design and drafted the manuscript. SR conceived the study, participated in study design, and drafted the manuscript. All authors read and approved the final manuscript.
